# Modelling Drug Delivery to the Small Airways: Optimization Using Response Surface Methodology

**DOI:** 10.1007/s11095-024-03706-1

**Published:** 2024-05-16

**Authors:** Hyunhong J. Min, Stephen J. Payne, Eleanor P. Stride

**Affiliations:** 1 Institute of Biomedical Engineering, Department of Engineering Science, University of Oxford, Oxford, UK; 2 Institute of Applied Mechanics, National Taiwan University, Taipie, Taiwan

**Keywords:** design of experiment, particle deposition model, particle size, pulmonary drug delivery, small airway targeting

## Abstract

**Aim:**

The aim of this *in silico* study was to investigate the effect of particle size, flow rate, and tidal volume on drug targeting to small airways in patients with mild COPD.

**Method:**

Design of Experiments (DoE) was used with an in silico whole lung particle deposition model for bolus administration to investigate whether controlling inhalation can improve drug delivery to the small conducting airways. The range of particle aerodynamic diameters studied was 0.4 – 10 µm for flow rates between 100 – 2000 mL/s (i.e., low to very high), and tidal volumes between 40 – 1500 mL.

**Results:**

The model accurately predicted the relationship between independent variables and lung deposition, as confirmed by comparison with published experimental data. It was found that large particles (~ 5 µm) require very low flow rate (~ 100 mL/s) and very small tidal volume (~ 110 mL) to target small conducting airways, whereas fine particles (~ 2 µm) achieve drug targeting in the region at a relatively higher flow rate (~ 500 mL/s) and similar tidal volume (~ 110 mL).

**Conclusion:**

The simulation results indicated that controlling tidal volume and flow rate can achieve targeted delivery to the small airways (i.e., > 50% of emitted dose was predicted to deposit in the small airways), and the optimal parameters depend on the particle size. It is hoped that this finding could provide a means of improving drug targeting to the small conducting airways and improve prognosis in COPD management.

**Supplementary Information:**

The online version contains supplementary material available at 10.1007/s11095-024-03706-1.

## Introduction

The small conducting airways are receiving increasing attention in chronic obstructive pulmonary disease (COPD) management. These airways, also known as the ‘silent zone’ of the lungs, have been historically overlooked because they account for only a tiny fraction of airway resistance (< 10%) in healthy lungs [1, 2] and thus thought to play only a minor role in airflow limitation in COPD patients. The lack of methods to measure the deterioration of small conducting airways has aggravated the situation. Spirometry, the primary method of measuring lung function, could not detect changes in the small conducting airways, especially in the early stages of COPD [3]. Thus, small conducting airways remained irrelevant in COPD management until a few decades ago. However, the finding that closures of small conducting airways are responsible for emphysematous destruction and increased peripheral airway resistance [4–6] in COPD patients redirected the focus of the research, and this is now an active field in COPD management.

Small conducting airways, also known as small tracheobronchial airways, are typically defined as those lacking cartilage and having an internal diameter of less than 2 mm [7]. These airways are located between the eighth generation of airways and the respiratory bronchioles. In COPD patients, tissues in the small airways exhibit chronic inflammation. The inflammation causes morphological changes in the cells. For instance, the airway walls are thickened due to squamous metaplasia and goblet cell hyperplasia, and inflammatory exudates obstruct the airway lumen [8]. These factors lead to reduced airway radius and, hence, airflow. The reduction in the small airway radius can further aggravate symptoms by premature closures of airways during expirations, leading to destruction in alveoli and a reduction in the number of acinar airways [9, 10].

Targeting therapy to small conducting airways, however, is challenging. A CFD simulation study has shown that conventional dry powder (e.g., Flovent Diskus) and metered-dose inhalers (e.g., Flovent HFA MDI) deliver less than 1% of the inhaled dose to small conducting airways [11]. Extrafine (i.e. < 1 μm diameter) particle inhalers have been shown to enable better lung deposition in the peripheral regions [12, 13] and improvements in symptoms in patients with severe COPD, e.g., reduction in exacerbation rate and improved lung function [14–16]. However, the optimal particle size of aerosols to effectively reach and deposit in the small airways has not been clearly defined [17].

Drug targeting in inhaled therapies involves modification of both drug particle characteristics and the inhalation pattern. Particle deposition in the upper and central airways is predominantly driven by inertial impaction [18]. Therefore, large particles (5 – 20 µm) and high flow rates favour deposition in the upper and central airways [19, 20]. Tidal volume also plays a role: an increase in tidal volume increases particle penetration and deposition in the deeper lungs and vice versa [21].

Various studies have attempted to optimize drug targeting in the lungs by changing inhalation patterns and/or particle characteristics [22–24]. Brand *et al*., 2005 [22] studied the effect of tidal volume and flow rate on particle deposition in peripheral lungs of cystic fibrotic patients using 2, 3, 4, and 5.5 µm monodisperse particles. Other *in vivo* human studies have investigated the effect of particle size on regional deposition [23, 24]. However, no studies (to the best of the authors’ knowledge) have examined the combined effect of tidal volume, flow rate, and particle diameter on deposition in the small conducting airways.

The use of a combination of Design of Experiment (DoE) and *in silico* whole lung particle deposition modeling can provide additional and valuable information that is difficult to obtain from studies involving *in vivo* or *in silico* whole lung particle deposition modeling alone. DoE is a statistical method of designing experiments to create a descriptive model. It has been used in various industries (e.g., aerospace [25], chemical synthesis [26], agriculture [27], and pharmaceutical manufacturing [28]) for parameter optimization. By leveraging both techniques, we aim to find the optimal tidal volume, flow rate, and particle diameter for targeted particle deposition in the small conducting airways. Bolus administration, as delivered by soft-mist inhalers, is assumed. Pressurized metered-dose inhalers (pMDI) produce a high initial particle velocity regardless of inhalation rate, so they are not considered here. It is hoped that the study will improve in the management of COPD and potentially enhance the quality of life for those affected by the disease.

## Methodology

### Description of the Lung Geometry

The airway model used for the present work was based on a deterministic multiple-path lung model [29, 30]. Computational Fluid Dynamics (CFD) was not utilized, hence specific airway geometry could not be included in the model. Nonetheless, research has shown that there is no significant difference in airway dimensions between healthy individuals (GOLD 0) and patients with mild COPD (GOLD 1) [31]. Therefore, the multiple-path lung model, which is created using data from healthy subjects, can be considered suitable for predicting lung deposition in individuals with mild COPD.

The tracheobronchial airway model used for the present work was based on a deterministic multiple-path lung model [29, 30]. The development of high-resolution CT imaging has facilitated non-invasive measurement of upper tracheobronchial airways. For example, Montesantos *et al*. (2013) measured tracheobronchial airway dimensions (e.g., diameter, length, and branching angle) in seven healthy subjects (n = 7) [31]. For his study, upper tracheobronchial airway dimensions (Generation 1–7) were taken from Montesantos *et al*. [31] and gravity angles from Raabe *et al*. [32]. For Generations 8 to 21 of the tracheobronchial airways, data from Koblinger (1985) were used to create a deterministic multiple path lung model [33]. The cross-sectional area ratio between parent and daughter airways was randomly assigned from the reported average and its distribution in Koblinger (1985). The diameter ratio of the major and the minor daughter airways was similarly randomly selected from its reported distribution and airway length was determined from the corresponding diameter-length correlation. The whole lung characteristics of the current model were compared to those of other lung models and experimental values, and are presented in our previous paper [34].

Instead of using a 3D airway model, this lung airway model utilized a binary tree data structure to represent the airway tree. Each node in the tree contained information about its parent and daughter nodes, and each connected airway had details about its necessary information for particle deposition efficiency. The process, known as tree traversal, is described in detail in Anjilvel and Asgharian [35].

### Deposition Calculations

It was assumed that no particles are present in the lungs prior to inhalation. Particles were assumed to be spherical and have a smooth and rigid surface and unit density. Airway walls were assumed to be rigid, and particle depositions were assumed upon contact. A steady flow rate and constant initial concentration were also assumed so that the particle concentration at the airway does not change as a function of time [36].

The effect of unsteady flow on particle deposition is most noticeable in the extrathoracic region, and thus its effect on particle deposition in the region was incorporated into the model by using an empirical equation [37]. The effect of unsteady flow on particle deposition in the airways beyond the extrathoracic region appears to be marginal [38], and thus unsteady flow is not expected to change the lung deposition significantly.

For particle deposition, several analytical equations were compared, and a combination of analytical equations that best fitted the *in vivo* data was selected [23, 34]. These were taken from Beeckmans (1965) [39], Ingham (1975) [40], and Landahl (1950) [41] for sedimentation, diffusion, and impaction deposition, respectively. Golshahi *et al*., [37] was used for extrathoracic deposition for the bolus administration model. Particles were assumed to be spherical, to have a smooth surface, to be rigid, and to have a unit density (i.e., 1 g/mL). Airway walls were also assumed to be rigid, and particle deposition was assumed to occur upon contact.

An analytical solution to inertial impaction was performed by calculating the relaxation time and stop distance of the particle in the bifurcating region and then solving them in the given geometry. Landhal, 1950 [41] assumes that the curvature of bifurcating airways follows a circular path but the path of the particle, due to its inertia, will deviate from that of the airway path. *r*_*c*_ is the curvature radius for the particle, and *r*_*0*_ is the curvature radius for the airway. At the end of the bending region, particles (*r*_*c*_) would have deviated a certain distance (*δ*) from the airway (*r*_*0*_), i.e., *δ* = *r*_*c-*_* r*_*0*_, and particles that were travelling at the top *δ* distance at the initial point of the bending region will deposit by the end of the bending region. This may be written as:

1 $$\mathrm\delta=r_c-r_0=\theta h\;\cdot\;St$$

where *θ* is the bending angle in radians,* h* is the airway's diameter, and *St* is the Stokes number.

Considering the circular cross-section circle:

2 $${\varepsilon }_{i}=1-\frac{2}{\pi }{\mathit{cos}}^{-1}\left(\theta St\right)+\frac{1}{\pi }\mathit{sin}\{2{\mathit{cos}}^{-1}\left(\theta St\right)\}$$

where *θ* is the bending angle in radians, *ε*_*i*_ is impaction deposition efficiency, and *St* is the Stokes number.

Sedimentation deposition, driven by the particle weight, occurs during the particle travel. In a simplified equation, it calculates the time over which particles travel through the airway (i.e., $t= {~}^{L}\!\left/ \!{~}_{U}\right.$), and then calculates the downward distance travelled during this time (i.e., $d={V}_{s} \times time$). Therefore, particles at distance *d* from the bottom at the proximal end would have travelled downwards and deposited by the distal end, and the deposition fraction may be represented as $\frac{d}{h}.$ Beeckmans [39] came up with semi-empirical equations based on particle deposition experiments in cylinders and was able to capture the re-entrance of particles into parent airways upon angulation. These equations incorporate the effect of gravity angle (*φ*), the orientation of airway (uphill/downhill), relaxation time (*τ*), and settling velocity (*V*) into a negative exponential term and calculate their effect on deposition probability:

3 $$\mathrm{For\;up\;hill\;flow},\;\mathrm{P\;(S)}= 1-\mathrm{e^{-\frac{4g\;L\;\uptau cos\;\varphi}{\pi D(U-V\;sin\;\varphi)}}}$$

4 $$\mathrm{For}\;\mathrm{down}\;\mathrm{hill}\;\mathrm{flow},\;\;\mathrm P\left(\mathrm S\right)=1-\mathrm e^{-\frac{4\mathrm g\;\mathrm L\;\uptau\;\cos\;\varphi}{{\pi{\mathrm{D}}}(\mathrm U+\mathrm V\;\sin\;\varphi)}}$$

where *P(S)* is sedimentation deposition probability, *g* is gravity, *L* is length of airway, *φ* is the gravity angle, *D* is diameter of airway, *U* is particle velocity along the airway and *V* is the settling velocity.

The diffusion equation [40] is derived by solving the steady-state mass diffusion equation for a cylindrical airway:

5 $${u}_{r}\frac{\partial c}{\partial r}+{u}_{z}\frac{\partial c}{\partial z}=D\left[\frac{1}{r}\frac{\partial }{\partial r}\left(r\frac{\partial c}{\partial r}\right)+ \frac{{\partial }^{2}c}{\partial {z}^{2}}\right]+q$$

where *c* is the concentration of particles per unit volume, *u* is the average particle velocity by advection, *D* is the diffusion coefficient, and *q* is the rate of formation of aerosols per unit volume of the flowing fluid. Particle position vectors in an airway are expressed in cylindrical coordinates, where *r* is the length of the vector on the airway cross-sectional plane and *z* is the length in the airway length direction. *φ* would have represented the angle of the vector on the cross-sectional plane, but particles are assumed to spread equally in all directions in this case, and hence it is not included.

Ingham [40] assumes that no additional aerosols are formed (i.e., *q* = *0*), and this is true for the current application. Movement of the particle in the direction *z* (or along airway length) by diffusion is negligible compared to their movement due to advection. Hence, $\frac{{d}^{2}c}{d{z}^{2}}=0$. Airflow is assumed to have zero velocity in the cross-sectional plane, i.e., *u*_*r*_ = *0*. *u* can be defined as $U(1-\frac{{r}^{2}}{{r}_{0}^{2}})$ in Poiseuille flow, where U is the centreline velocity, and the r_0_ is the radius of the airway. Equation (5) can be described as below:

6 $$U(1-\frac{{r}^{2}}{{r}_{0}^{2}}) \frac{dc}{dz}=D\left[\frac{1}{r}\frac{d}{dr}\left(r\frac{dc}{dr}\right)\right]$$

Applying the boundary conditions *c* = *c*_*0*_ at *z* = *0* for all *r*, and *c* = *0* at *r* = *r*_*0*_ for *z* > *0* (i.e., the same concentration of particles enters at all points on its surface and particles deposit when they touch the airway wall), Eq. (6) can be solved using a perturbation method [40], yielding:

7 $$\text{P}_\text{D}=1- 0.819{e}^{-14.63\Delta }-0.0976{e}^{-89.22\Delta }-0.0325{e}^{-228\Delta }-0.0509{e}^{-125.9{\Delta }^{^{2}\!\left/_{\kern-.6pt{3}}\right.}}$$

where $\Delta =\frac{DL}{4U{R}^{2}}$. *D* is the diffusion coefficient, *L* is the airway length, *R* is the airway radius, and *U* is the average centreline air velocity.

Bolus dispersion was modelled using the method from Hofmann *et al*., [42]. The model includes the effect of (1) mixing in the extrathoracic region, (2) convective mixing in conducting airways, (3) mixing at airway bifurcations, and (4) mixing of residual and new air in the alveoli. The bolus model was validated against *in vivo* lung deposition data for polydisperse dry powder inhalers [43–45]. The result is available in [34]. MATLAB R2022b (The MathWorks, Inc.) was used for the *in silico* lung particle deposition model.

### Response Surface Methodology

This study used response surface methodology (RSM) to characterize the relationship between tidal volume, flow rate, particle diameter, and small conducting airway deposition (or small tracheobronchial deposition). The regression analysis was then used to find the optimal parameters that improved deposition. The optimal values were investigated using the bolus model within the following ranges: 100 – 2000 mL/s for flow rate, 40 – 1500 mL for tidal volume, and 0.4 – 10 µm for particle diameter. Respiratory time was fixed at 10 s. The ranges of flow rate and tidal volume were chosen to represent physiologically relevant breathing conditions. The particle diameter range reflects that used in commercially available dry powder inhalers.

**Fig. 1:**
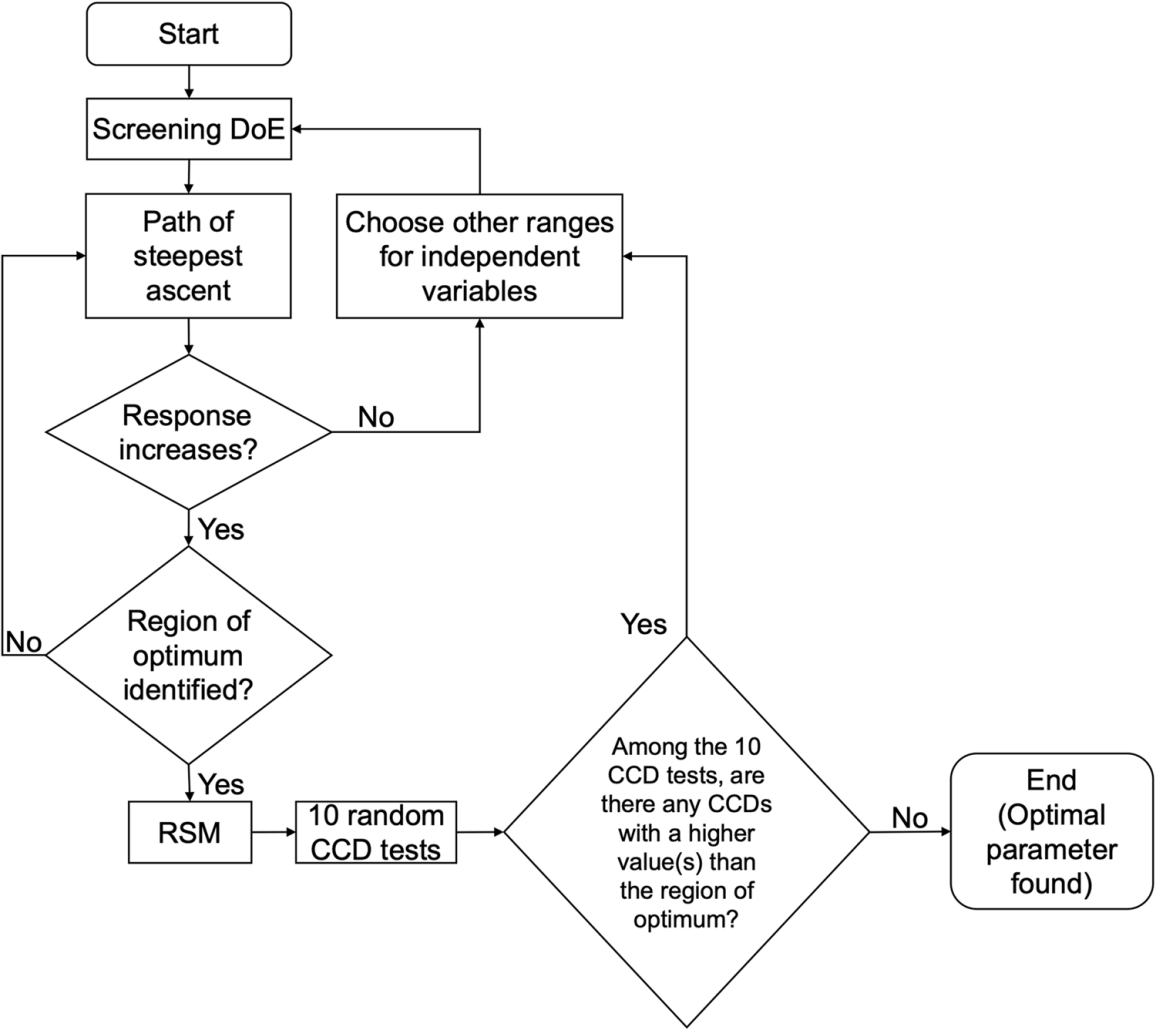
Shows the steps used to find the optimal parameters for targeted delivery in small tracheobronchial airways. It began with the initial screening step. A minimum number of simulations was used to find a linear relationship between the three variables (particle diameter, flow rate, and tidal volume) and the deposition. The linear relationship derived from the screening step was used for the method of steepest ascent step. The variables were changed in the direction of increasing the deposition. This step allowed us to find the region of the optimum. CCD model was created inside the region. The model was used to find the optimal parameters for the drug targeting. The flow chart ends with a double-checking step. Ten CCD models were created at random points inside the studied ranges to confirm that there are no other regions of the optimum.

A screening step was first performed (Fig. 1) to characterize the relationship between the independent variables (i.e., particle diameter, flow rate, and tidal volume) and the response (i.e., small tracheobronchial deposition) in a simple first-order manner within the selected parameter range. The screening step used a fractional factorial (2^3–1^) design. Low inhaled flow rate (i.e., 500—1000 mL/s), low tidal volume (i.e., 200 – 400 mL), and extrafine dry powder inhalers (i.e., 0.4 – 1.0 µm) have been reported to improve deposition in distal airways [22], and thus these parameter ranges were selected for the screening step.

The linear equation derived from the screening step was then used to determine the direction of change for each independent variable to increase the response (Fig. 1). This is called the method of steepest ascent. It helps to find the region of the optimum, which is the parameter range that includes the optimal parameter set. The independent variables were increased or decreased in equal intervals. They continued until the response started to drop, indicating the region of the optimum.

Central composite design (CCD) was then used to develop a descriptive second-order model inside the region of the optimum point (Fig. 1). The model was described by linear terms (${x}_{i}$), quadratic terms (${x}_{i}^{2}$), cross-product interaction terms (${x}_{i}{x}_{j}$), intercept (${\beta }_{0}$) and the error term ($\varepsilon$). To avoid overfitting, terms that have insignificant contributions to the response were deleted based on ANOVA analysis. Terms with *p*-values greater than 0.10 in ANOVA were eliminated using backward elimination.

The quadratic model was assessed for accuracy and predictability using adjusted R^2^ and predicted R^2^ values [46]. Contour plots and factorial plots were used to visualize and examine the relationship between independent variables and the response. The optimal parameter conditions were found, and the predicted responses were validated using the lung deposition model.

Lastly, ten random CCD simulations were conducted within the studied range (namely, 100 – 2000 mL/s for flow rate, 40 – 1500 mL for tidal volume, and 0.4 – 10 µm for particle diameter). This checked for any other region of optimum that may have a high small tracheobronchial deposition. RSM and its statistical analysis were conducted using Minitab® Version 20 (Minitab LLC) software. 

## Results and Discussion

### Response Surface Methodology

The screening step revealed that small tracheobronchial deposition increased with an increase in particle diameter and decreased with an increase in flow rate and tidal volume (Supplementary Information Figure S1). In the method of steepest ascent step, flow rate and tidal volume were decreased by 50 mL/s and 20 mL, respectively. At the same time, particle diameter increased by 0.1 in log-10 scales (Supplementary Information Figure S1). Incrementally increasing particle diameter in log-10 scales allowed more data points in the smaller particle diameters (1 – 5 µm), where changes in response are proportionally larger. It showed the change in deposition with incremental changes in each independent variable. CCD was thus set up around the region where small tracheobronchial deposition is highest to analyze the relationship (Supplementary Information Figure S1).

The CCD model accurately described the relationship, as suggested by the high adjusted R^2^ value (88.63%), but the difference between adjusted R^2^ and predicted R^2^ values (> 10%) suggested possible overfitting (Table I). It was, however, later confirmed in the validation study (Table II) that the response prediction by RSM is in good agreement with the whole lung deposition simulation data.

**Table I:** A quadratic model equation for small tracheobronchial deposition (Small TB) is presented. A, B, and C are particle diameter, flow rate, and tidal volume, respectively. Superscript 2 on the alphabet represents the quadratic terms, and two alphabets represent interaction terms (i.e., AB is the interaction term for A and B). Terms were removed from the equation using the backward elimination method for *p*-value > 0.10

Deposition region	Equation	R^2^ (adj)	R^2^ (pred)
Small TB	$y=-186.1 + 52.80\mathrm{ A }+ 2.29\times {10}^{-1}\mathrm{ B }+ 2.11\mathrm{ C}- 4.63 {{\text{A}}}^{2}- 9.70\times {10}^{-3} {{\text{C}}}^{2}- 7.09\times {10}^{-2}\mathrm{ AB}$	88.63%	74.46%

**Table II:** Optimal parameter sets for high deposition percentage in small tracheobronchial are given. The predicted deposition percentage using the CCD Model (‘Predicted’) is compared to the in silico lung particle deposition model (‘Validation). The in silico model values given are the averages of 20 samples (n = 20), and 95% Confidence Interval (C.I.) is given. ‘Predicted’ and ‘Validation’ values were found not to be significantly different (p < 0.05, 2-way t-test)

Regional deposition	Optimal parameter set			Deposition percentage (%)	
	Diameter (µm)	Flow Rate (mL/s)	Tidal volume (mL)	Predicted (95% C.I.)	Validation (95% C.I.)
Small TB	4.9	100.0	109.1	64.6% (51.9 – 77.3%)	53.6% (38.9 – 65.2%)
	2.0	500.0	109.1	55.5% (38.0 – 72.5%)	42.0% (38.9 – 45.2%)

Ten more CCD models were created at random points within the studied ranges, but no other optimal region was found in the studied ranges. This suggests that the region found by the method of steepest ascent is the only region relevant for delivery to the small tracheobronchial airways (Supplementary Information Figure S1).

### Targeted Delivery to Small Tracheobronchial Airways

**Fig. 2:**
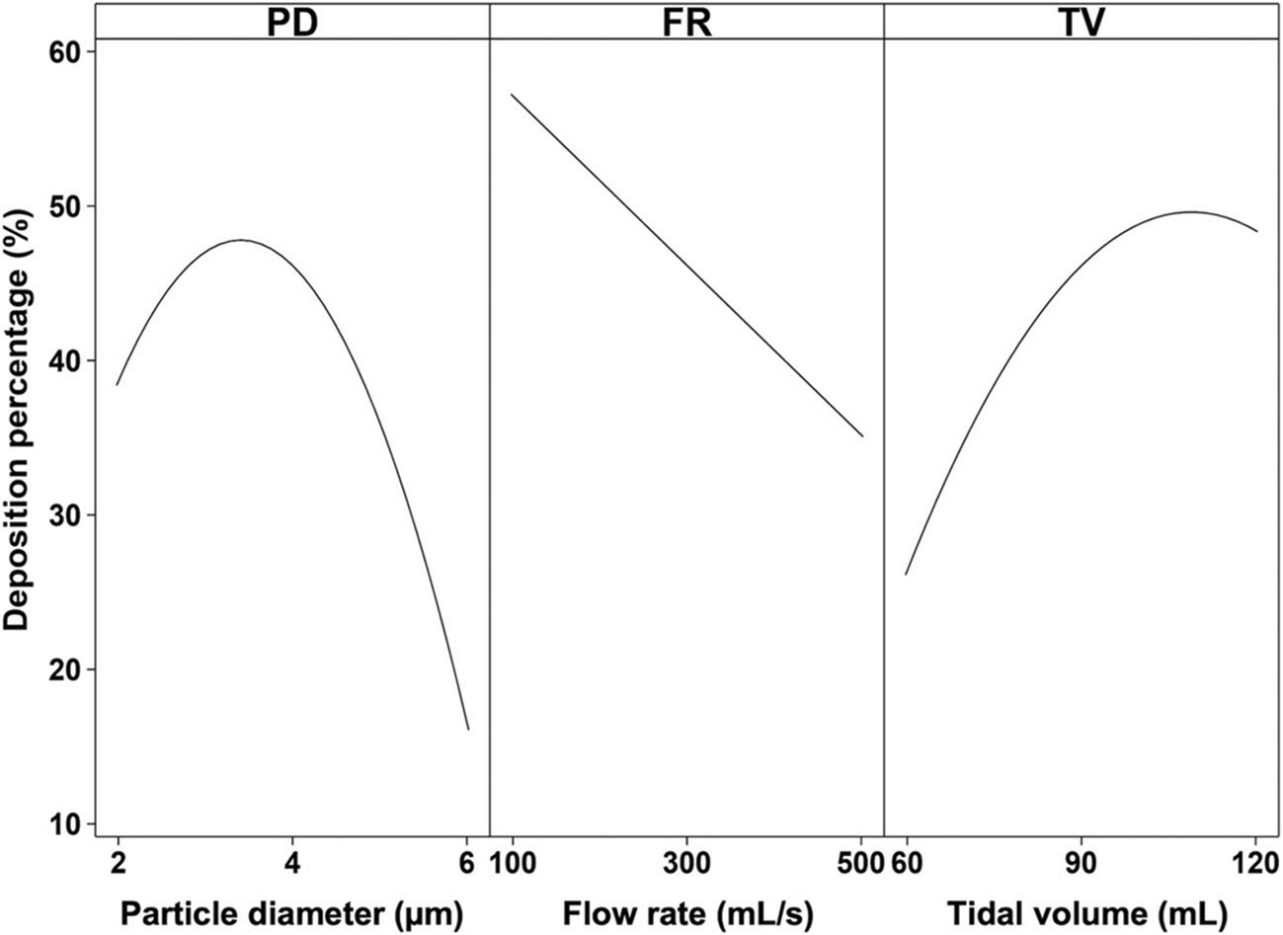
The predicted effects of particle diameter, flow rate (FR), and tidal volume (TV) on small tracheobronchial deposition.

As expected from the literature, deposition in the small tracheobronchial airways was found to be affected by tidal volume, particle size, and flow rate (Fig. 2). Tidal volume and particle diameter had a negative quadratic relationship with deposition, suggesting that local maxima and hence potential optimal conditions exist.

**Fig. 3:**
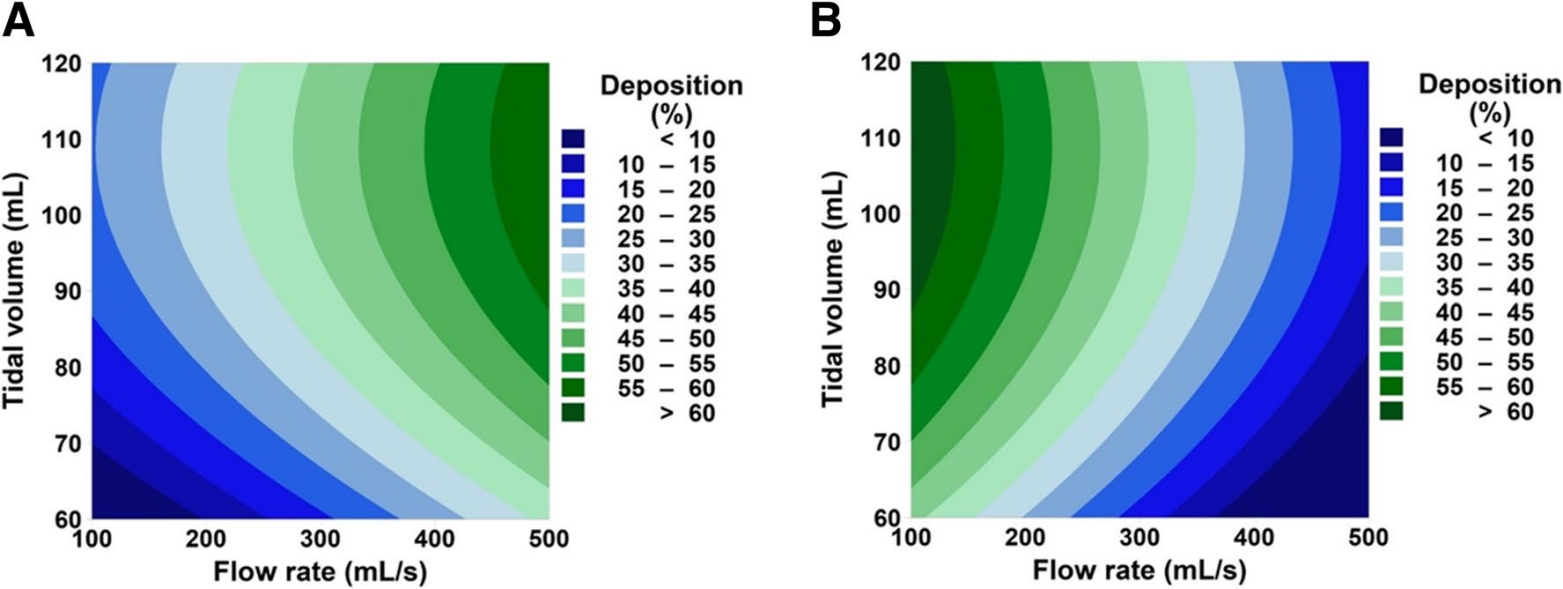
Contour plots showing the effects of tidal volume and flow rate on small tracheobronchial deposition for monodisperse aerosols having particle diameters of (**A**) 2 µm and (**B**) 4.9 µm. The graphs show that small tracheobronchial deposition can vary from below 10% to above 50%, depending on the tidal volume and flow rate.

Small tracheobronchial airway deposition was found to be highly dependent on tidal volume and flow rate. Figure 3A and B show the effect of flow rate and tidal volume on deposition of 2 µm and 4.9 µm monodisperse aerosol particles, respectively. Both particle sizes showed the highest deposition at 109.1 mL tidal volume. The flow rate, on the other hand, affected the two particle sizes differently. It was found that a higher flow rate (500 mL/s) was favourable for aerosols of 2 µm diameter and vice versa for monodisperse 4.9 µm aerosols.

**Fig. 4:**
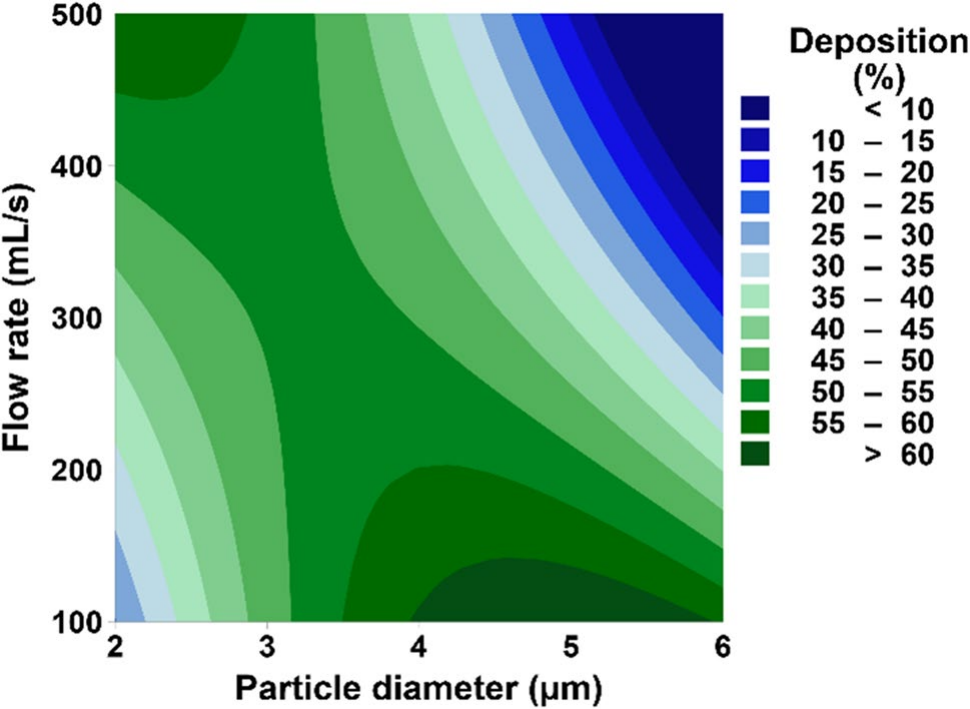
A contour plot shows the effects of particle diameter and flow rate on small tracheobronchial deposition. Tidal volume is set at 109.1 mL.

Figure 4 shows that the optimal flow rate for small tracheobronchial deposition varies with particle size. The larger particles (i.e., 4 – 6 µm) increased deposited in the airways at slower flow rates (i.e., around 100 mL/s), whereas the smaller particles (i.e., 2 – 3 µm) increased the deposition at higher flow rates (i.e., around 500 mL/s). Large particles (i.e., 4 – 6 µm) at slow flow rates facilitate increased particle penetration into the small tracheobronchial airways because the slow flow rate reduces deposition in the extrathoracic and large tracheobronchial regions. On the other hand, smaller particles (i.e., 2 – 3 µm) deposit in the small airways better at higher flow rates because of inertial impaction in the small tracheobronchial airways.

The optimal sets of parameters for targeting small conducting airways may however change depending on various factors, such as the disease state and age. The airway model is based on healthy lungs, which should accurately represents the lung airways of mild COPD patients. However, as COPD progresses into moderate or severe states, the number of distal airways reduces and airways begin to narrow [47]. Despite the fact that several clinical studies have found that the disease state of COPD does not change lung deposition and its deposition pattern [48–50], the authors believe that these factors may change the optimal breathing and particle parameter for targeting the small conducting airways. Firstly, the location of stenosis in the lung airways can affect the lung particle deposition differently. For instance, stenosis in the upper airways can increase deposition in the region, and thus reduce the available particles in the small conducting airways [51]. Secondly, reduced number of distal airways in certain regions of the lungs can selectively reduce air flow and thus particle deposition in those specified parts of the lungs. Therefore, it is suspected that the severity of COPD will affect the lung particle deposition, and thus the optimal set of parameters would change.

The optimal parameters were found using the desirability function approach, incorporated within ‘Response Optimization’ function of Minitab®. It found two optimal parameter sets (Table II) suggesting that large aerosols sized 4.9 µm in diameter could target the small tracheobronchial airways when inhaled at a flow rate of 100 mL/s and with a tidal volume of 109.1 mL; and that particles with a relatively small diameter (2 µm) could target the small tracheobronchial airways when inhaled at 500 mL/s of flow rate and 109.1 mL of tidal volume.

It was previously reported that commercial inhalers deliver less than 1% of the emitted dose to the small conducting airways, which are typically inflamed in COPD [11]. Controlling aerosol size has been suggested as a means of improving drug delivery to the region, and inhalers with extrafine particles (i.e., < 1 µm) have been reported to improve symptoms of COPD [17, 52, 53]. There is, however, no direct evidence of increased drug delivery to the small conducting airways nor direct proof of reduction in inflammation in the small conducting airways. The improvement in symptomatic outcomes could be a result of increased deposition in the alveolar airways and increased systemic exposure.

In contrast to the currently accepted notion that particle size control is crucial for targeting the small conducting airways, this study suggests that drug targeting may be achieved by more simply controlling the tidal volume and flow rate. Figure 4 shows that a wide range of particle sizes from 2 to 6 µm can achieve a high deposition percentage (> 50%) if flow rate and tidal volume are controlled. Similarly, Fig. 3, shows that the change in flow rate and tidal volume can reduce deposition percentage from above 50% to below 10%. The findings suggest that inhalers with different aerosol sizes could successfully target the small conducting airways by controlling the flow rate and tidal volume of COPD patients.

### Practical Considerations and Limitations

In this study, targeted delivery to the small tracheobronchial airways was found to require fine control of tidal volume and flow rate. Such fine control of breathing, however, is unachievable without a device that controls the entire inhalation maneuver of patients. For example, the AKITA inhalation system (Activaero, Gemünden, Germany) is a computer-controlled compressor that stores and analyses subjects’ breathing patterns. It controls the tidal volume and flow rate by applying positive air pressure. AKITA has successfully controlled subjects’ breathing characteristics and reduced flow rate and tidal volume variability [54].

Unfortunately, the AKITA inhalation system is not yet compatible with inhalers for bolus administration (e.g., pMDI, DPI, or soft-mist inhalers) and there are no other systems currently available. The lack of inhalation control systems may be due to poor incentives for development. The market for the inhalation control systems is limited to clinical trials, and thus the market size is insufficient to incentivize new development. This study, however, shows that controlling tidal volume and flow rate is critical for targeting the small conducting airways. If inhalation systems were found to improve prognosis in COPD patients, they might become a central part of COPD management.

If found to be efficacious, the treatment for small conducting airways would complement the current therapy rather than a replacement. Deposition in the small conducting airways alone would not improve the prognosis of COPD patients because other regions of the lung airways, such as large conducting airways and respiratory airways, also play essential roles in COPD management. For instance, large conducting airways determine lung function tests such as Forced Expiratory Volume in 1 s (FEV_1_), Forced Vital Capacity (FVC), and FEV_1_/FVC [55]. The acini are responsible for gas exchange [56]. Thus, the treatment for small conducting airways would be an add-on to the current regimen of COPD management, which may help to improve small airway obstructions and thus patient prognosis.

There are, however, some important limitations for practical implementation. The first is that the device-flow rate interaction adds complexity to optimising drug delivery with dry powder inhalers. The negative peak pressure (which controls the flow rate) determines the emitted dose and the emitted particles' mass median aerodynamic diameter (MMAD). To effectively target the small conducting airways, a slow inspiratory flow rate is required, typically below 500 mL/s. However, some dry powder inhaler devices, such as Turbohaler®, may experience a reduction in emitted dose of up to 20% at this flow rate [57]. This reduction can ultimately lead to a decrease in the total dose delivered to the small conducting airways. One potential solution to the issue of high peak negative pressure required for near 100% emitted dose is the development of low-volume dry powder inhaler devices [58, 59]. These devices, which were initially designed to deliver dry powders to preterm infants with nRDS who have low inspiratory flow rates, could be used in combination with an inhalation control system to effectively deliver powders to COPD patients at low inspiratory flow rate.

Another possible limitation is that increasing the complexity of the treatment regimen can result in poor adherence. Over 50% of COPD patients show poor adherence to treatment after five years [60].

Effective patient education and behavioural assistive tools may help to address poor adherence. The authors are optimistic that the integration of digital technology with the inhalation control system can provide a promising solution to enhance adherence to this complex treatment regimen [61]. Further research in this field can yield valuable insights and ideas to further improve patient outcomes.

The current lung deposition model assumes that the aerosols have no initial velocity. Hence, the aerosols have the same velocity as the air. This means the findings apply to dry powder inhalers and possibly soft-mist inhalers but not pressurized meter-dose inhalers. In future studies, particle–fluid dynamics of different inhalers can be incorporated into the model to study the effect of inhaler device on small tracheobronchial deposition.

## Conclusion

This study used the DoE methodology to find the optimal breathing and particle characteristics for targeting the small conducting airways. The model developed was compared to experimental data and was found to accurately describe the relationship between lung regional particle deposition and three independent variables (tidal volume, flow rate, and particle size). The model found that all particles between 2 and 6 µm can sufficiently target the small conducting airways (i.e., > 50% of emitted dose) when tidal volume and flow rate are controlled. This study suggests that tidal volume and flow rate are critical for drug targeting. Thus, the development of an inhalation control system that is compatible with DPI, pMDI, or soft-mist inhalers may help to achieve drug targeting to the small conducting airways.

## Supplementary Information

Below is the link to the electronic supplementary material.

Supplementary file1 (DOCX 2946 KB)
