# Supplementary material for: Modelling Drug Delivery to the Small Airways: Optimization Using Response Surface Methodology
Source: Pharm Res. 2024 May 16;41(6):1139–48. doi: 10.1007/s11095-024-03706-1 (PMC11525259; doi:10.1007/s11095-024-03706-1)
Supplement: Supplementary file 1 — Supplementary file1 (DOCX 2946 KB) [file 11095_2024_3706_MOESM1_ESM.docx]

# Supplemental Information for:

**Modelling drug delivery to the small airways: optimization using Design of Experiments (DoE) methodology**

**Hyunhong J. Min^1^, Eleanor P. Stride ^1^, and Stephen J. Payne ^1, 2^**

**^1^** Institute of Biomedical Engineering, Department of Engineering Science, University of Oxford, Oxford, UK

^2^ Institute of Applied Mechanics, National Taiwan University, Taiwan

**Supplementary Information 1.**


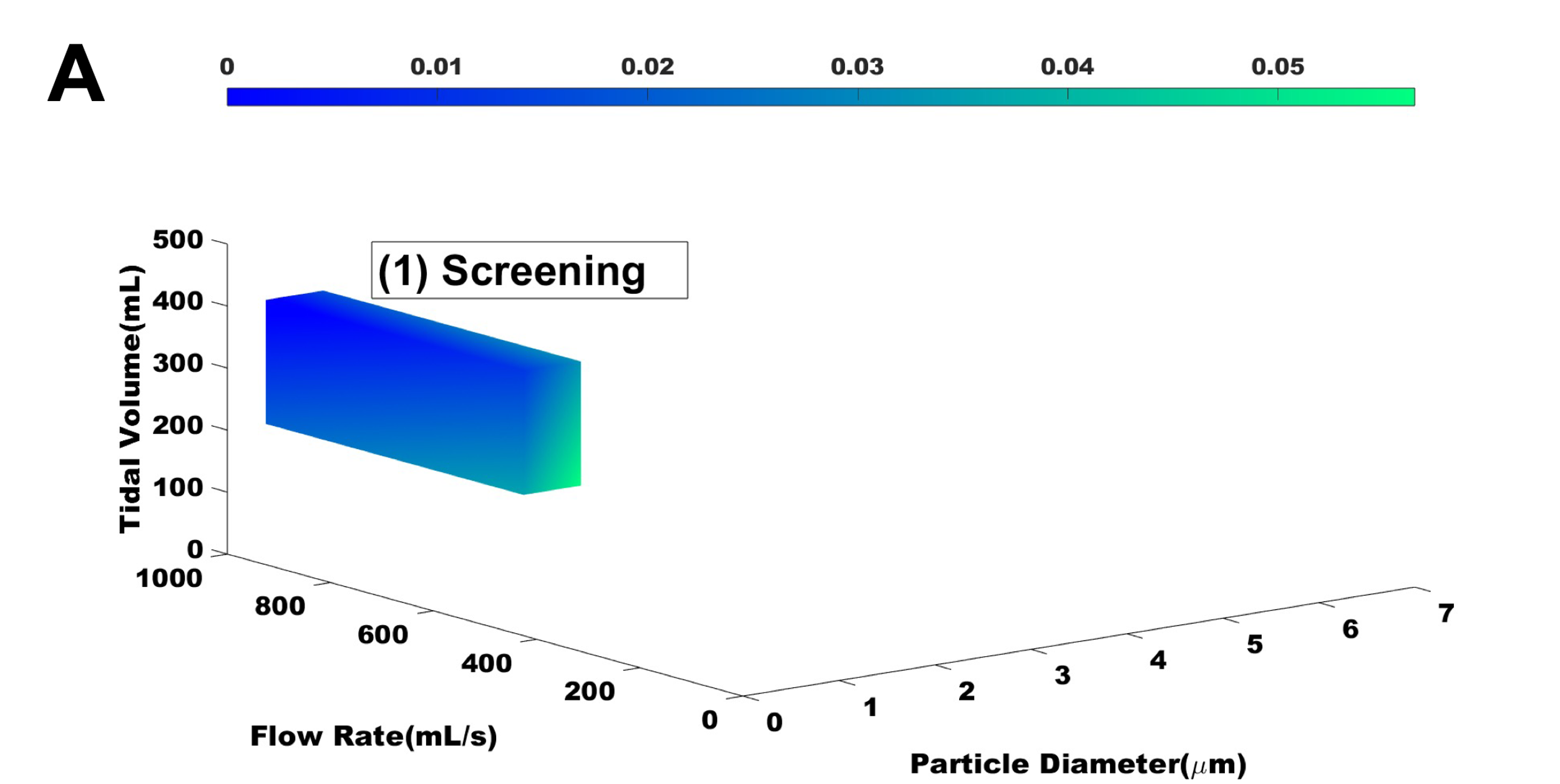


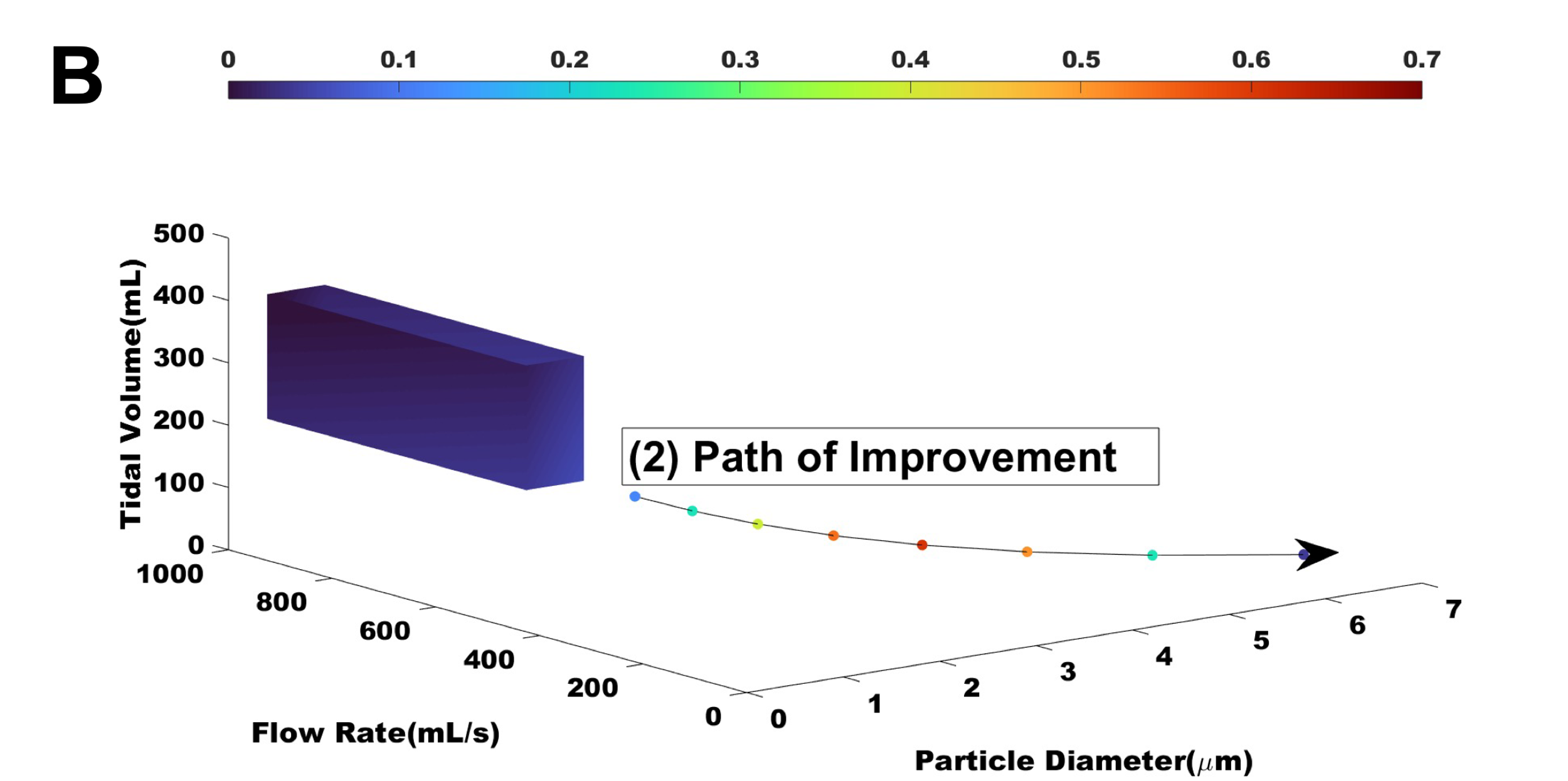


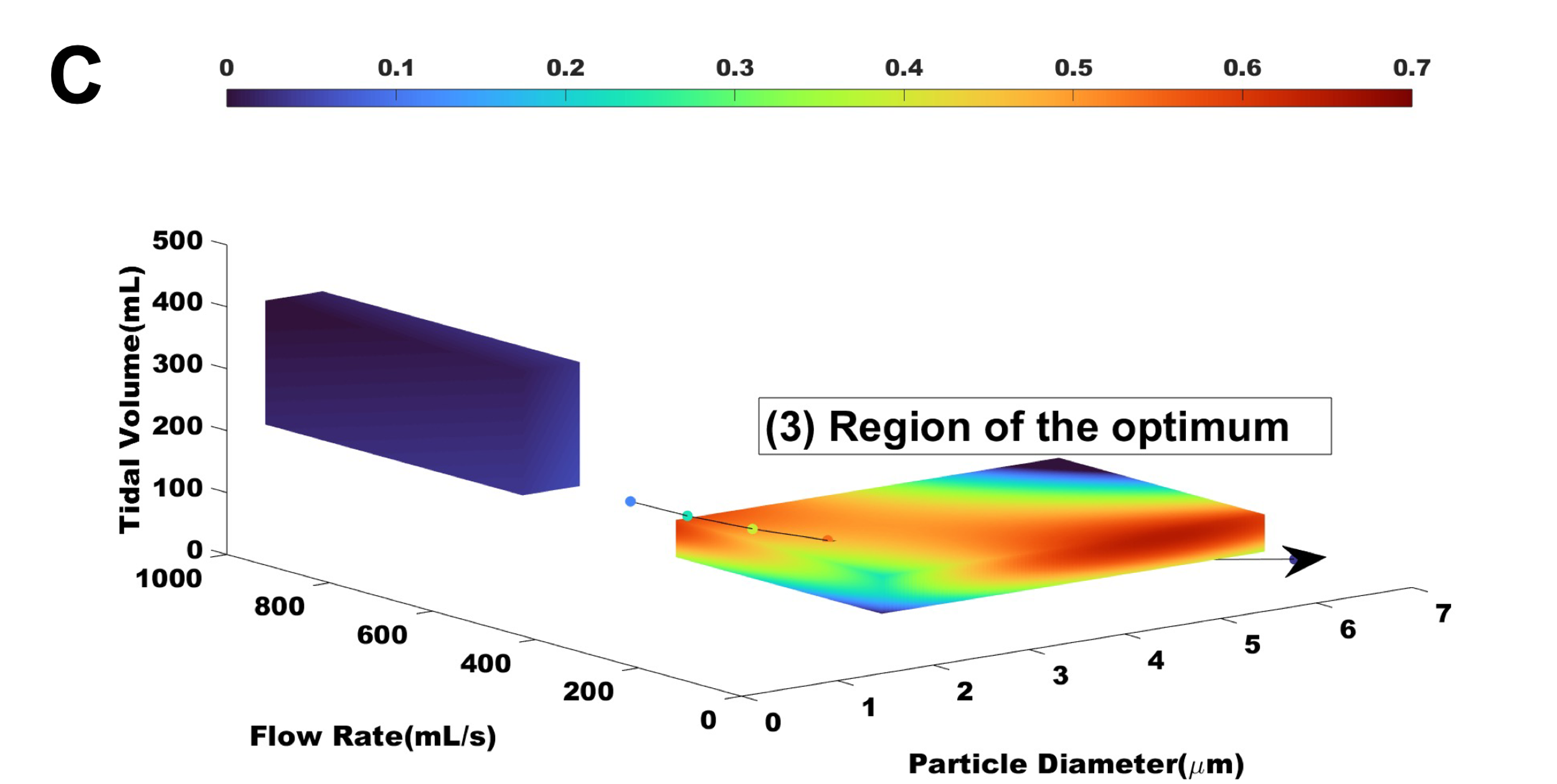

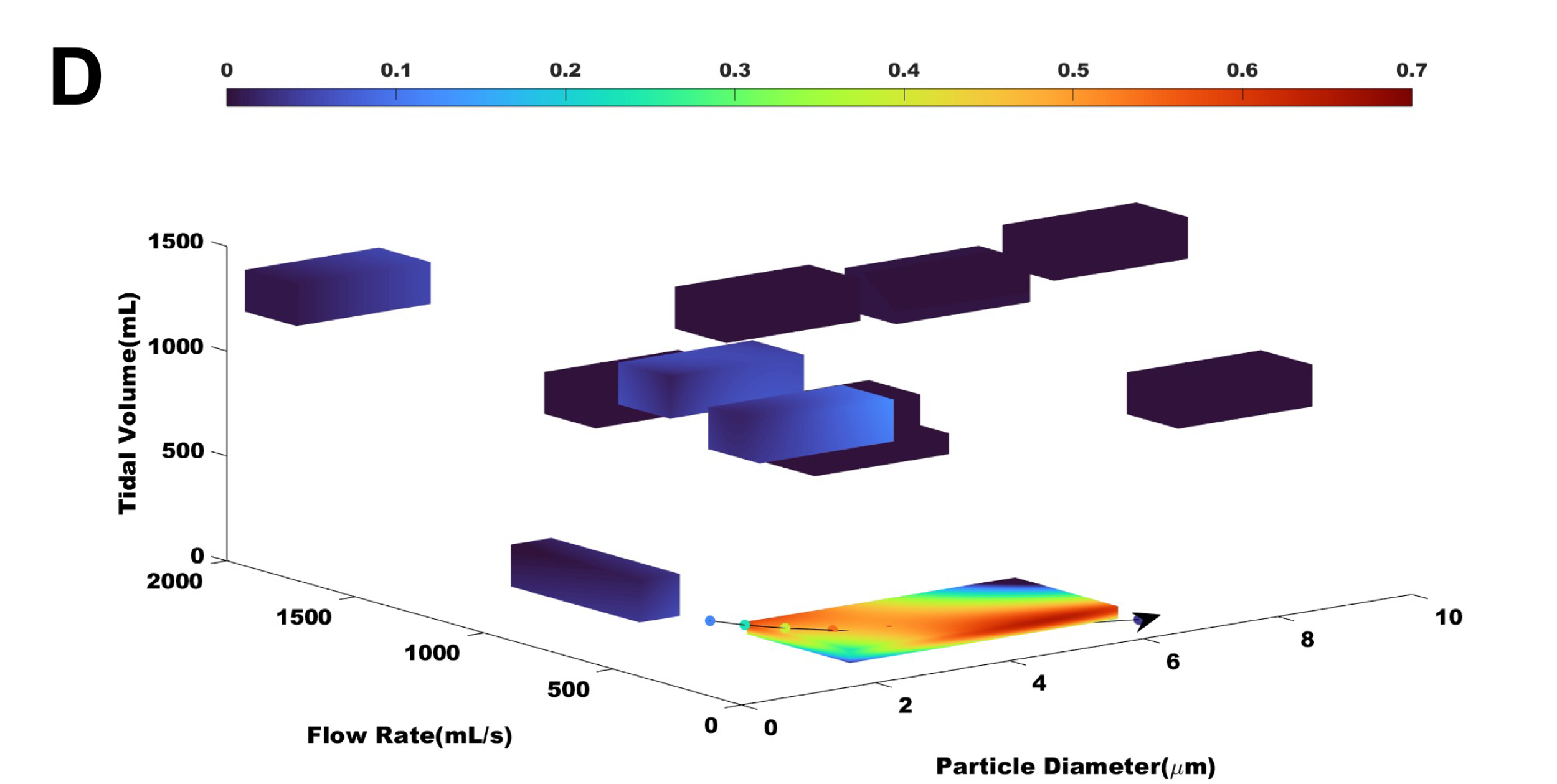


Figure S1. shows the steps used to find the optimal parameters for targeted delivery in small tracheobronchial airways. (A) shows the initial screening step. A minimum number of simulations was used to find a linear relationship between the three variables (particle diameter, flow rate, and tidal volume) and the deposition. (B) is the method of steepest ascent step. The variables were changed in the direction of increasing the deposition. This step allowed us to find the region of the optimum. (C) shows a CCD model that was created inside the region. The model was used to find the optimal parameters for the drug targeting. (D) shows a double-checking step. Ten CCD models were created at random points inside the studied ranges to double-check that there are no other regions of the optimum. The color bar represents the deposition percentage (%).
